# Global Healthcare Resource Efficiency in the Management of COVID-19 Death and Infection Prevalence Rates

**DOI:** 10.3389/fpubh.2021.638481

**Published:** 2021-04-29

**Authors:** Marthinus C. Breitenbach, Victor Ngobeni, Goodness C. Aye

**Affiliations:** ^1^Department of Economics, University of Pretoria, Pretoria, South Africa; ^2^National Treasury of the Republic of South Africa, Pretoria, South Africa

**Keywords:** pandemic, COVID-19, death rates, infection rates, recoveries, data envelopment analysis, healthcare systems efficiency, technical efficiency

## Abstract

The scale of impact of the COVID-19 pandemic on society and the economy globally provides a strong incentive to thoroughly analyze the efficiency of healthcare systems in dealing with the current pandemic and to obtain lessons to prepare healthcare systems to be better prepared for future pandemics. In the absence of a proven vaccine or cure, non-pharmaceutical interventions including social distancing, testing and contact tracing, isolation, and wearing of masks are essential in the fight against the worldwide COVID-19 pandemic. We use data envelopment analysis and data compiled from Worldometers and The World Bank to analyze how efficient the use of resources were to stabilize the rate of infections and minimize death rates in the top 36 countries that represented 90% of global infections and deaths out of 220 countries as of November 11, 2020. This is the first paper to model the technical efficiency of countries in managing the COVID-19 pandemic by modeling death rates and infection rates as undesirable outputs using the approach developed by You and Yan. We find that the average efficiency of global healthcare systems in managing the pandemic is very low, with only six efficient systems out of a total of 36 under the variable returns to scale assumption. This finding suggests that, holding constant the size of their healthcare systems (because countries cannot alter the size of a healthcare system in the short run), most of the sample countries showed low levels of efficiency during this time of managing the pandemic; instead it is suspected that most countries literally “threw” resources at fighting the pandemic, thereby probably raising inefficiency through wasted resource use.

## Introduction

Since it first emerged in China in late December 2019, the new coronavirus (COVID-19) spread to nearly every country of the world (1). Within 7 months, it had spread to 215 countries and regions. At the time of producing this paper, on November 11, 2020, 52 million people were known to be infected (2), and ~1.3 million deaths had been recorded since the outbreak. Countries adopted pandemic spread mitigating interventions referred to as non-pharmaceutical interventions (NPIs), such as social distancing, testing and contact tracing, case isolation, and public hygiene at an unprecedented scale (3). Without a proven vaccine or cure, non-pharmaceutical interventions including social distancing, testing, wearing of masks, and contact tracing are essential to end the worldwide COVID-19 (4).

Even with these drastic NPI interventions, the spread of the pandemic exploded, especially with surges in contagion experienced in countries like Italy, France, the UK, and the USA. This put immense strain on the availability of especially intensive care unit facilities, doctors, and nurses, and the efficiency of healthcare systems was also put under the spotlight. What we learn from recent experiences in the fight against this deadly disease from countries like South Korea is that accessibility to healthcare services can significantly reduce the number of deaths (5). Moreover, Sarkar et al. (4) used a mathematical model to demonstrate that the elimination of the ongoing SARS-CoV-2 pandemic is possible by combining restrictive social distancing and contact tracing. They concluded that the accurate course of the epidemic heavily depends on how and when quarantine, isolation, and precautionary measures are enforced. This is also supported by Breitenbach et al. (6). According to Khajanchi and Sarkar (7), in the absence of specific antivirals or vaccines, mathematical modeling plays an important role in better understanding the disease dynamics and in designing strategies to control the rapidly spreading infectious disease. Samui et al. (8) used a compartmental mathematical model to predict and control the transmission dynamics of COVID-19 pandemic in India with epidemic data up to April 30, 2020. They computed a basic reproduction number, R0, of 1.7. This showed a substantial outbreak of COVID-19 in India. Their model predicted that, for about 60 days, the peak will be higher for COVID-19 infections in India and after that the curve will plateau, but the coronavirus disease will persist for a long time.

It is for this reason and the impact of COVID-19 on society and the global economy that the efficiency of healthcare systems needs to be thoroughly examined. This could inform appropriate policy responses and adequately prepare health systems to respond better to future pandemics. Our study is different from typical compartmental models as we address the issue of macro-efficiency of public healthcare systems by applying data envelopment analysis (DEA), a non-parametric and mathematical model adept to estimate the technical efficiency of public healthcare systems. We also use extensive data compiled from Worldometers (2) and the World Bank (9–11). Specifically, we analyze the efficient use of available resources to stabilize the rate of infections and minimize the case fatality rates in the top 36 selected countries representing 90% of global infections and deaths in 220 countries as of November 11, 2020. Our contribution to the literature is 2-fold: first, this paper is the first to model the technical efficiency of countries in dealing with the COVID-19 pandemic by modeling death rates and infection rates as undesirable outputs and, second, by modeling comparative scenarios to test the accuracy of our model. Modeling contagion curves and estimating efficiency rates may contribute to policies and strategies to assist public healthcare systems in the fight against this pandemic. However, the role of media is invaluable in educating the population about the dangers of the pandemic and the importance of using NPIs. This can potentially change the publics' behavior and affect the implementation of individuals' intervention and control strategies (12).

## Literature Review

DEA has been applied extensively to compare the efficiency of healthcare facilities within countries and between countries, and we briefly deal with some of that literature here. We do not deal with the literature on country studies because our paper compares efficiency between countries. For literature on efficiency studies among different healthcare facilities within a country, see, for example, Ngobeni et al. (13), Campanella et al. (14), Alhassan et al. (15), Jarjue et al. (16), Chowdhury et al. (17), Gannon (18), Marschall and Flessa (19), Akazili et al. (20), Masiye (21), Zere et al. (22), and Kirigia et al. (23, 24).

Although healthcare is one of the most popular areas of application for DEA (25), DEA studies on healthcare systems worldwide are still limited. For example, Bhat (26) used DEA to measure the impact of financial and institutional arrangements on national healthcare system efficiency in 24 OECD countries. Lo Storto and Goncharuk (27) applied DEA to measure the technical efficiency of 32 European (EU) countries. Afonso and St Aubyn (28) used a two-stage DEA to estimate a semi-parametric model of the healthcare systems in 30 OECD countries for the years 1995 and 2003. De Cos and Moral-Benito (29) estimated alternative measurements of efficiency using DEA and stochastic frontier analysis between 1997 and 2009 to ascertain the most important determinants of healthcare efficiency across 29 OECD countries. Hadad et al. (30) compared the healthcare system efficiency of 31 OECD countries with two model specifications, one including inputs under management control and the other including inputs beyond management control. Kim and Kang (31) used a bootstrap DEA to estimate the efficiency of healthcare systems in a sample of 170 countries.

Although the choice of inputs is similar in these studies, outputs selection depends mostly on the purpose of the research. For example, Gonzalez et al. (32), in a cross-sectional study, measured the technical and value efficiency of health systems in 165 countries. They used expenditure on health and education as inputs and data on healthy life expectancy and disability adjusted life years as health outcomes. Examining the efficiency in healthcare services delivery to the population, Bhat (26) uses the number of populations aged 0–19, 20–64, and 65 years or older as outputs. Santos et al. (33) examine the efficiency of countries in preventing the mother-to-child HIV transmission and used the number of pregnant women tested for HIV and the number of HIV pregnant women receiving antiretroviral drugs as outputs.

DEA studies for new settings such as the recent COVID-19 outbreak may however need to introduce new outputs. Shirouyehzad et al. (34) uses DEA to analyze the efficiency of contagion of COVID-19 and focus on the number of deaths and recoveries as outcomes. Breitenbach et al. (6) analyze the 31 most infected countries during the first 100 days since the outbreak of the COVID-19 coronavirus for the efficiency in containing the spread of the virus and focus on flattening the curve as the main output. Empirical work pivots mostly on healthcare system performance based on technical efficiency calculated as a ratio of some quality-of-life variable as an output and physical health resources or expenditure on health as inputs. The inputs mostly used were expenditure, doctors, and nurses, while the outputs were discharge or recovery, prevalence, and mortality rates. In this paper, we use tests, doctors, and nurses as physical inputs and health spending as financial input in managing the COVID-19 pandemic. As outputs, we use case fatality (deaths) and infection prevalence rates.

## Methodology

In this paper, we use the variable returns to scale (VRS) approach reported by Gavurova et al. (35) and developed in 1984 by Banker, Charnes, and Cooper (BCC model) to allow for consideration of scale efficiency analysis. Envelopment in DEA refers to the ability of the efficiency production frontier to tightly enclose the production technology (input and output variables). According to Cooper et al. (36) and McWilliams et al. (37), DEA was developed in a microeconomic setting and applied to firms to measure the efficiency of converting inputs into outputs. In the analysis of public institutions, firms are replaced by the more encompassing decision-making units (DMU). DEA is therefore an appropriate method of computing the efficiency of institutions employing multivariate production technologies. Aristovnik (38) and Martić et al. (39) distinguish between input minimization and output maximization DEA models. The former determines the quantity of inputs that could be curtailed without reducing the prevailing level of outputs, and the latter expands the outputs of DMUs to reach the production possibility frontier while holding the inputs constant. However, the selection of each orientation is study specific. In this paper, we select input minimization orientation, as the objective of the study is to measure the efficiency of resources used (minimized inputs) at prevailing health output levels (recovery, death, and infection rates). It is unwise to select an output maximization dispensation as it would be tantamount to maximizing death and infection rates as desirable outputs alongside the recovery rate. When undesirable outputs are an inevitable by-product in the production process, the input minimization orientation is selected as the preferred DEA [also see You and Yan (40)].

According to Taylor and Harris (41), DEA is a comparative efficiency measurement tool that evaluates the efficiency of homogeneous DMUs operating in similar environmental conditions, for example, DMUs dealing with COVID-19 and where the relationship between inputs and outputs is unknown. We follow Joumard et al. (42) to treat the whole healthcare system in a given country as a DMU in order to analyze the healthcare system at the aggregate level. We also adopt the VRS methodology in this study because of heterogeneity among the DMUs in terms of factors like country size and income. In terms of the DEA methodology, the current study uses the BCC model, with the ratio of DMUs being four times the combined number of inputs and outputs to ensure the stability of the efficiency results.

### Modeling Undesirable Outputs

DEA models have found increasing use in efficiency analysis applications where at least one output in the production process is an undesirable output, e.g., pollution. There is considerable research published on the undesirable aspects of production outputs. However, You and Yan (40) have found that the economic implications and the suitability of DEA models incorporating the undesirable outputs should be carefully considered as the results may either under- or overstate efficiency if modeled incorrectly.

The first way that undesirable outputs are dealt with in the traditional DEA model is to ignore the undesirable output (43–46). It is not, however, appropriate to ignore the reality of, e.g., pollution during production since undesirable outputs and desirable outputs are generated simultaneously in the production process. Dyckhoff and Allen (47) dealt with undesirable outputs by modeling them as inputs. However, treating undesirable outputs as inputs fails to reflect the true production process. There is a specific production technology that links inputs to outputs, and taking an undesirable output as an input in the production process leads to misspecification and misinterpretation, for example, when modeling pollution as an input using an output-oriented measure, ecological inefficiencies remain undetected. Golany and Roll (48) suggested a data transformation approach where an undesirable output is converted into a “normal” output by a monotonic decreasing function. The undesirable outputs (carbon and nitrogen emissions) are treated as normal outputs by taking their reciprocals. Although the pollutant is treated as output, the scale and intervals of the original data get lost, and the problem with zero values is that it does not have a reciprocal value. The linear monotonic decreasing transformation was suggested by Seiford and Zhu (49). A sufficiently large positive scalar β_*i*_ is added to the reciprocal additive transformation of the undesirable output *i* so that the final values are positive for each DMU_k_. This model is criticized for its invariance to data transformation within the DEA model (45, 46). Färe et al. (50) treats undesirable factors in a non-linear DEA model based on the weak disposability of undesirable outputs (51). Weak disposability assumes that, to reduce undesirable outputs, it is costly because simultaneously it increases the inputs or decreases desirable outputs (52). It tends to increase the desirable and undesirable output concurrently. Regardless of the form of transformation, as long as the final value of undesirable output included in the DEA calculation remains positive, it increases the efficiency of the DMU. An undesirable output should bring either a negative or positive impact to the performance of the DMU; therefore, it is not appropriate for the undesirable output to solely favor the efficiency score.

After comparing the performance of the models discussed above, You and Yan (40) developed the ratio model, which outperformed all five of these models developed for dealing with undesirable outputs. We therefore opted to adopt the ratio model for the current paper. The ratio model is different from the previous approaches in that the undesirable output is aggregated in a ratio form with the desirable output.

From the conventional BCC DEA model and assuming that there are *R* DMU_*r*_ (*r* = l, 2,…, *R*) that convert *m* inputs to *n* outputs, DMU_*k*_ is one of the *R* DMU*s* being evaluated. It is further assumed that DMU_*k*_ consumes *m* inputs $X_{t}^{k}$ (*i* = 1, 2, . . . , *m*) to produce *n* outputs $Y_{j}^{k}\;$(*j* = 1, 2, . . . , *n*), and all these outputs are assumed to be desirable. The measure of efficiency of DMU_*k*_ is then obtained by:

min θ subject to

(1) $$\begin{array}{r} \overset{R}{\underset{r=1}{\sum }}\lambda_{r}X_{i}^{r}-\theta X_{i}^{k}+s_{i}^{-}=0\;i\;=\;1,\;2\;,\ldots ,\;m \end{array}$$

(2) $$\begin{array}{r} \overset{R}{\underset{r=1}{\sum }}\lambda_{r\;}Y_{j}^{r}-s_{j}^{+}=\;Y_{j}^{k}\;j\;=\;1,\;2,\;\ldots ,\;n \end{array}$$

(3) $$\begin{array}{rcl} \overset{R}{\underset{r=1}{\sum }}\lambda_{r}\;=1\\ \lambda_{r,\;}{\;s}_{i}^{-},\;s_{j}^{+}\; & \geq & 0\;r=1,\;\ldots ,\;R \end{array}$$

where DMU_*r*_ = the *r*th DMU, *r* = 1,2, . . . , *R*; DMU_*k*_ = the *k*th DMU being evaluated; $X_{i}^{r}$, $Y_{j}^{r}=$ the inputs and outputs of every DMU_*r*_; *i* = 1, 2, . . . , *m*, j = 1,2, . . . , *n*; θ = the efficiency of DMU_*k*_*;* λ_*r*_ = the dual variable corresponding to the other inequality constraint of the primal;

$s_{i}^{-},\;s_{j}^{+}$ = the slack variables that turn the inequality constraint into an equal form; $\lambda_{r,\;}^{{}^{*}}\;s_{i}^{{-}^{*}}$, $s_{j}^{{+}^{*}}\;=\;$the optimal solutions when the relative efficiency of DMU_*k*_ is θ^*^ = 1 and $s_{i}^{{-}^{*}}=\;s_{j}^{{+}^{*}}=\;0$.

In the ratio model, the undesirable output and desirable output are defined as $O_{q\;}^{-}\;\left(q=1.\;2,\;\ldots ,\;n_{1}\right)$ and $O_{p}^{+}\;\left(p=1,\;2,\;\ldots ,\;n_{2}\right)$, respectively (*n*_1_+ *n*_2_ = *n*). For DMU_*k*_, the undesirable outputs $O_{q}^{-}(q=1,\;2,\;\ldots ,\;n_{1}$) are treated as a new variable ψ_*k*_, which is called the penalty parameter and is written as:

(4) $$\begin{array}{r} \psi_{k}=\;\rho_{1}O_{1k}^{-}+\ldots +\;\rho_{n1}O_{n1k}^{-} \end{array}$$

where ψ_*k*_ = penalty parameter for DMU_k_; ρ_*q*_ = the penalty for individual undesirable output (*q* = 1, 2, …, *n*_1_); $O_{q\;}^{-}=\;$the undesirable output (*q* = 1, 2, …, *n*_1_). Since ρ_*q*_ is the penalty charged for producing the outputs, the ψ_*k*_ obtained from problem (10) gives a measure of the total monetary value of undesirable outputs. From the definition of ψ_*k*_, the greater the amount of undesirable output, the greater is the value of the penalty parameter. Furthermore, the respective value of ρ_*q*_ is associated with the individual undesirable output; therefore, ρ_*q*_ has the same value for every DMU. With this model, desirable and undesirable outputs can relate to one another, regardless of a disagreement in units. With the new approach of treating the undesirable outputs in Equation (10), the desirable output *p* (*p* = 1, 2, …, *n*_2_) of DMU_k_ in the ratio model is modified as:

(5) $$\begin{array}{r} Y_{\rho }^{{}^{\prime }}=\;\frac{1}{\psi_{k}}O_{p}^{+},\;\left(p=1,\;2,\;\ldots ,\;n_{2}\right) \end{array}$$

where $O_{p}^{+}=$ the desirable output (*p* = 1, 2, …, *n*_2_), and $Y_{\rho }^{{}^{\prime }}$ = the modified output (*p* = 1, 2, …, *n*_2_ ).

The ratio model computes desirable and undesirable outputs as fractions, where undesirable output $O_{q}^{-}$ is the denominator and desirable output $O_{p}^{+}$ is the numerator. Here the value of the output is interpreted as a ratio of desirable to undesirable output. Using ratios provides a simple and easy way to expose the impact of undesirable outputs in a DEA. The ratio form of the DEA model can satisfy the restrictions of the conventional DEA, which the output variable states must be a positive value. Moreover, the ratio form provides a more distinct way for the desirable and undesirable output to describe the presence of an undesirable output on DMU efficiency.

In order to check the stability of our model results, we ran three different model specifications and compared the results. In model I, we use the number of tests and number of doctors and nurses as physical inputs, health expenditure as financial input, and the ratio of recoveries to infection rates as output (ratio of desirable to undesirable output). In model II, we use the number of tests and number of doctors and nurses as physical inputs, health expenditure as financial input, and the ratio of recoveries to death rates as output (ratio of desirable to undesirable output), and in model III, we use the number of tests and number of doctors and nurses as physical inputs, health expenditure as financial input, and the number of recoveries as output. In model III, we therefore ignore the undesirable outputs (43–46). Although it is not good to ignore the undesirable outputs of the rate of new infections and death rates, we do this in order to compare the difference that the inclusion of the undesirable outputs in our model has on the efficiency scores.

### Data

Our data were gathered from different sources. The COVID-19-related data (i.e., infected cases, recovered cases, deaths, and number of tests) were extracted from extensive data compiled from Worldometers (2). The aggregated data on doctors and nurses per 100,000 of the population and healthcare expenditure were obtained from world development indicators provided by the World Bank (9–11). As reported earlier, we analyze the efficient use of available resources to stabilize the rate of infections and minimize the case fatality rates in the top 36 selected countries (see Table A1) representing 90% of global infections and deaths in 220 countries as of November 11, 2020.

Some descriptive statistics of the variables reported in Table 1 indicate that our sample countries have, on average, resources of nearly seven doctors and nurses per 1,000 of the population, a budget of about 8% of gross domestic product (GDP) and 200,850 tests per one million of the population for its healthcare system. The number of infected cases and deaths from COVID-19 over the study period averaged more than 1,295,120 and 32,821, respectively, and the mean number of people recovering from the infection was around 974,487 persons. Assuming that the whole healthcare system is mobilized to fight the COVID-19 outbreak, how efficient was the mobilization of resources? This issue is analyzed with our DEA model, and the results are reported in the next section.

**Table 1:** Descriptive statistics and variables used in the model.

**Variables**	**No. of observations**	**Unit**	**Mean**	**Standard deviation**	**Minimum**	**Maximum**
**Physical Inputs**						
No. of Tests	36	per million of the population	200,849.78	159,220.81	15,033.00	541,193.00
No. of Doctors & Nurses	36	per 1,000 of the population	7.00	5.00	1.00	22.00
**Financial Input**						
Health Expenditure	36	% of GDP	8.00	3.00	3.00	17.00
**Desirable output**						
Recovery Rate	36	No. of People	974,486.67	1,844,065.41	30,504.00	8,023,412.00
**Undesirable output**						
Death Rates	36	No. of People	32,820.67	50,619.93	1,174.00	245,989.00
Infection Rates	36	No. of People	1,295,119.31	2,265,355.91	175,711.00	10,575,373.00

*Authors' calculations based on Worldometers (2) and The World Bank (9–11)*.

## Results

**Figure 1:**
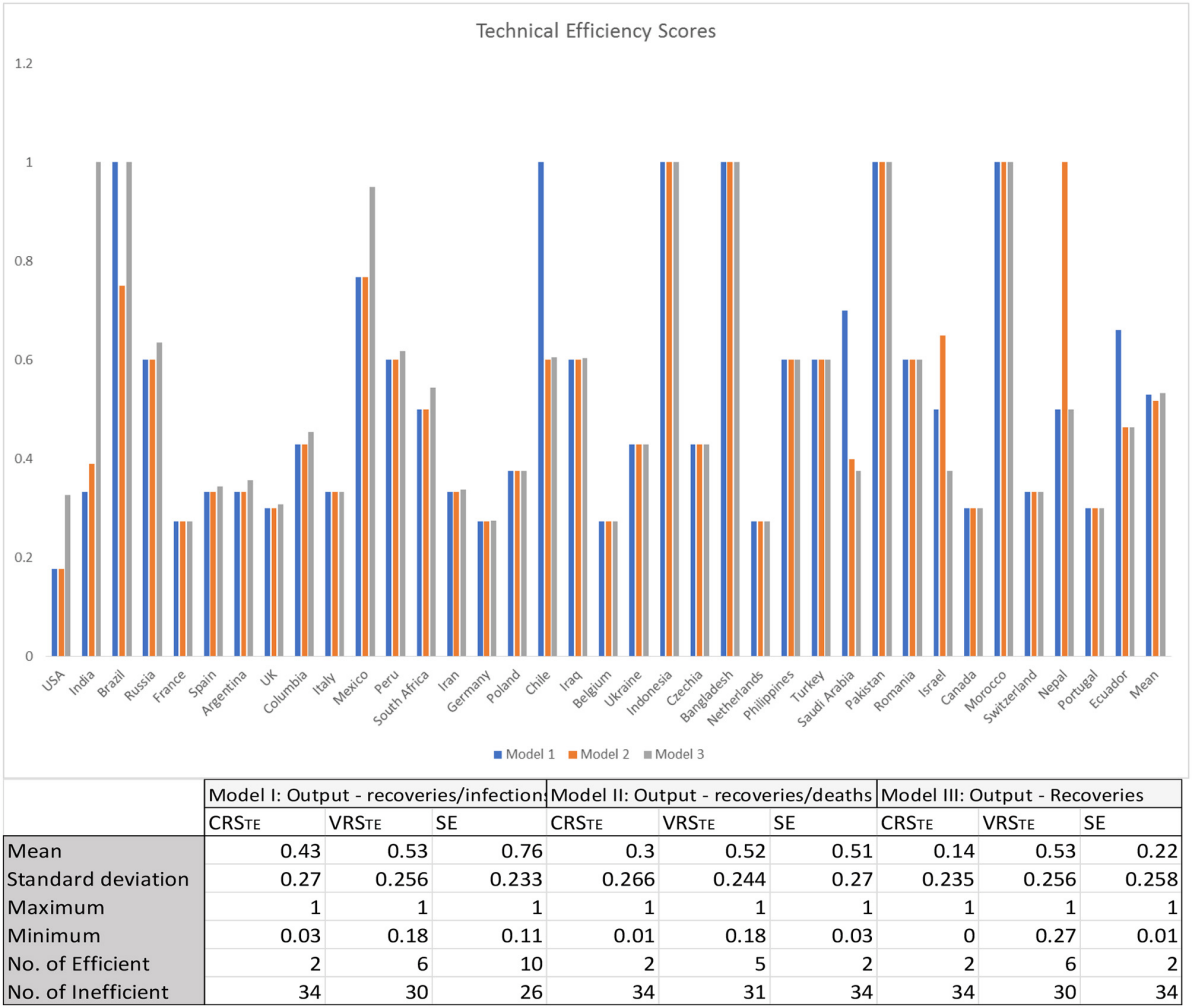
Constant returns to scale and variable returns to scale efficiency scores of global healthcare systems. CRST_E_ represents technical efficiency under constant returns to scale assumption, VRST_E_ represents technical efficiency under variable returns to scale assumption, and SE represents scale efficiency.

The results of the three model variants are graphically illustrated in Figure 1, and the results are presented in Table 1 (Table A1). As intimated earlier in this paper, it is important to consider the VRS technical efficiency scores motivated by the differences in the size of healthcare systems globally, particularly between large developed economies and small less-developed economies. The VRSTE scores are almost identical across the three model variants. This points to two things: first, the inclusion of undesirable outputs in our model (variants I and II) does not have any material impact on the mean technical efficiency of country healthcare systems and, second, it points to the stability of our results across the three model variants. For the sake of simplicity, we therefore discuss only the results reflected in model I, where our physical inputs were the number of tests/million of the population and number of doctors and nurses per 100,000 of the population and our financial input healthcare expenditure as a percentage of GDP and our output recoveries/infections. Under the CRS assumption, there were only two efficient healthcare systems in dealing with COVID-19, *viz*., Bangladesh and Pakistan. When the VRS assumption is considered, the figure rises as expected, in this case to six, with the addition of Brazil, Chile, Indonesia, and Morocco.

These differences, regarding the full sample of 36 countries, are statistically significant under a Mann–Whitney–Wilcoxon's test (*Z* = 5.271*, p* = 0.001). It indicates the role of scale efficiency in our analysis because it is the objective of global healthcare systems to achieve the optimal technical combination of the inputs to produce the outputs, but their scales (sizes) are not optimal yet. Although 21 of the 36 countries in our sample are operating under increasing returns to scale, the technical combination of inputs to produce the existing output is still not optimal. Six of the 36 countries operate under decreasing returns to scale (see the Table A1), suggesting that they can double their inputs without doubling their output. These countries could therefore rationalize their healthcare resources/inputs by downsizing (using resources/inputs more efficiently) and, thereby, improving the technical efficiency, while the outputs can still stay the same. At first glance, it is often difficult to envisage a country with a large undesirable output to be technically efficient. Brazil, for example, has a very high number of infections and deaths, yet our DEA results show that Brazil is technically efficient and lies on the efficiency frontier. To gain further insight into this number and the associated DEA efficiency scores, it is helpful to compare inputs and outputs of a benchmark country like Brazil relative to that of other countries. We have done this in Table 2.

**Table 2:** Inputs and outputs relative to the benchmark country (Brazil).

**Country**	**VRSTE**	**Expenditure (% of GDP)**	**Doctors & nurses/100,000**	**No. of tests**	**Infections**	**Deaths**	**Recoveries**
Brazil	1	4	4	102,766	5,701,283	162,842	5,964,344
USA	0.18	17	14	484,227	10,575,373	245,989	6,603,470
France	0.27	11	14	279,353	1,829,659	42,207	131,920
Germany	0.27	11	17	278,886	710,265	11,912	454,800
Belgium	0.27	11	14	458,403	507,475	13,561	30,504
**Comparison with Brazil**							
USA/Brazil		4.25	3.5	471.19%	185.49%	151.06%	110.72%
France/Brazil		2.75	3.5	271.83%	32.09%	25.92%	2.21%
Germany/Brazil		2.75	4.25	271.38%	12.46%	7.32%	7.63%
Belgium/Brazil		2.75	3.5	446.06%	8.90%	8.33%	0.51%

*Calculated from Table A1 results*.

For example, in comparison to Brazil, the USA spends 4.25 times more as a percentage of GDP on healthcare, has 3.5 times more doctors and nurses per 100,000 of the population, and had 471% more COVID-19 tests performed relative to Brazil, yet it did not succeed to contain its undesirable outputs (infections are 185% higher and deaths are 151% higher than Brazil) even though it performed well in the area of the good output—recoveries. This result clearly explains the relatively low VRS technical efficiency scores of the USA, France, Germany, and Belgium in Table 2, which could be linked to the specific policy responses of the selected countries. For example, evidence now suggests that the UK failed to fight the COVID-19 outbreak by following a “herd immunity” approach (53), and the USA was very slow to act against COVID-19 (54).

## Conclusions

This paper examined the efficiency of 36 healthcare systems (which represent 90% of cases globally) in managing the COVID-19 pandemic, given their resource constraints. We use a novel DEA approach, developed by You and Yan (40), which accounts for both desirable outputs (recovered cases) and undesirable outputs (infections and deaths), and our results indicate that the average efficiency of global healthcare systems in managing the COVID-19 pandemic is very low, with only six efficient systems out of a total of 36 under the variable returns to scale assumption. This finding suggests that, holding constant the size of their healthcare systems (because countries cannot alter the size of a healthcare system in the short run), most of the sample countries could not improve their efficiency during this time of managing the pandemic; instead it is suspected that most countries literally “threw” resources at fighting the pandemic, thereby probably raising inefficiency through wasted resource use. Inefficient countries could learn best practices of managing pandemics from the efficient countries in the sample, most being developing countries. This indicates to the global health sector that it is less about health resource endowments but more about the efficiency of using the available resources. The study also showed that, without pharmaceutical interventions like vaccines, the prevailing healthcare resources and NPIs used in combating major pandemics like COVID-19 appear to help fewer countries. Therefore, the healthcare sector should invest more in proactive than reactive management of pandemics, for example, through continuous research and development on preventative medication. The study is constrained in several ways. The DEA results are heavily dependent on the selection of analytical variables. Therefore, a different set of indicators may lead to a different collection of results. The credibility and accuracy of statistics used also affect the results of the models—data of the pandemic is getting more refined over time. This study adds to the literature on modeling the efficient use of resources in world healthcare systems with the inclusion of undesirable outputs. The methodology that we developed can, at any time, be replicated as new data becomes available as the pandemic progresses or when new pandemics develop.

## Data Availability Statement

The original contributions presented in the study are included in the article/supplementary material, further inquiries can be directed to the corresponding author/s.

## Author Contributions

All authors listed have made a substantial, direct and intellectual contribution to the work, and approved it for publication.

## Conflict of Interest

The authors declare that the research was conducted in the absence of any commercial or financial relationships that could be construed as a potential conflict of interest.

## Appendix

**Table A1:** Analytical variables and efficiency scores.

**Studied units**	**Model I**				**Model II**				**Model III**				
**DMU #**	**Country**	**CRS efficiency score**	**VRS efficiency score**	**Scale**	**Type of scale**	**CRS efficiency score**	**VRS efficiency score**	**Scale**	**Type of scale**	**CRS efficiency score**	**VRS efficiency score**	**Scale**	**Type of scale**
1	USA	0.12	0.18	0.67	IRS	0.07	0.18	0.39	IRS	0.29	0.33	0.89	DRS
2	India	0.33	0.33	1.00	–	0.38	0.39	0.96	DRS	1.00	1.00	1.00	–
3	Brazil	0.83	1.00	0.83	DRS	0.40	0.75	0.54	IRS	1.00	1.00	1.00	–
4	Russia	0.47	0.60	0.78	IRS	0.37	0.60	0.62	IRS	0.18	0.64	0.29	IRS
5	France	0.03	0.27	0.11	IRS	0.01	0.27	0.04	IRS	0.01	0.27	0.03	IRS
6	Spain	0.22	0.33	0.67	IRS	0.11	0.33	0.33	IRS	0.07	0.34	0.19	IRS
7	Argentina	0.33	0.33	1.00	–	0.16	0.33	0.48	IRS	0.16	0.36	0.46	IRS
8	UK	0.20	0.30	0.67	IRS	0.07	0.30	0.22	IRS	0.05	0.31	0.17	IRS
9	Columbia	0.43	0.43	1.00	DRS	0.22	0.43	0.50	IRS	0.16	0.45	0.34	IRS
10	Italy	0.15	0.33	0.44	IRS	0.04	0.33	0.13	IRS	0.03	0.33	0.08	IRS
11	Mexico	0.67	0.77	0.87	IRS	0.11	0.77	0.14	IRS	0.40	0.95	0.42	IRS
12	Peru	0.60	0.60	1.00	–	0.21	0.60	0.35	IRS	0.15	0.62	0.24	IRS
13	South Africa	0.50	0.50	1.00	–	0.30	0.50	0.61	IRS	0.13	0.54	0.24	IRS
14	Iran	0.26	0.33	0.78	IRS	0.07	0.33	0.22	IRS	0.09	0.34	0.27	IRS
15	Germany	0.18	0.27	0.67	IRS	0.15	0.27	0.55	IRS	0.03	0.27	0.10	IRS
16	Poland	0.17	0.38	0.45	IRS	0.15	0.38	0.41	IRS	0.03	0.38	0.07	IRS
17	Chile	0.67	1.00	0.67	DRS	0.30	0.60	0.49	IRS	0.09	0.61	0.15	IRS
18	Iraq	0.60	0.60	1.00	–	0.37	0.60	0.62	IRS	0.09	0.60	0.15	IRS
19	Belgium	0.03	0.27	0.11	IRS	0.01	0.27	0.03	IRS	0.00	0.27	0.01	IRS
20	Ukraine	0.24	0.43	0.55	IRS	0.16	0.43	0.36	IRS	0.03	0.43	0.07	IRS
21	Indonesia	0.95	1.00	0.95	IRS	0.44	1.00	0.44	IRS	0.23	1.00	0.23	IRS
22	Czechia	0.29	0.43	0.67	IRS	0.32	0.43	0.75	IRS	0.03	0.43	0.06	IRS
23	Bangladesh	1.00	1.00	1.00	–	1.00	1.00	1.00	–	0.43	1.00	0.43	IRS
24	Netherlands	0.18	0.27	0.67	IRS	0.13	0.27	0.46	IRS	0.02	0.27	0.07	IRS
25	Philippines	0.60	0.60	1.00	–	0.46	0.60	0.77	IRS	0.08	0.60	0.14	IRS
26	Turkey	0.60	0.60	1.00	–	0.27	0.60	0.45	IRS	0.05	0.60	0.09	IRS
27	Saudi Arabia	0.42	0.70	0.60	DRS	0.33	0.40	0.83	IRS	0.04	0.38	0.10	IRS
28	Pakistan	1.00	1.00	1.00	–	0.82	1.00	0.82	IRS	0.16	1.00	0.16	IRS
29	Romania	0.47	0.60	0.78	IRS	0.23	0.60	0.38	IRS	0.03	0.60	0.05	IRS
30	Israel	0.42	0.50	0.83	DRS	0.63	0.65	0.97	IRS	0.03	0.38	0.07	IRS
31	Canada	0.27	0.30	0.89	IRS	0.09	0.30	0.30	IRS	0.02	0.30	0.05	IRS
32	Morocco	0.89	1.00	0.89	IRS	0.88	1.00	0.88	IRS	0.02	1.00	0.02	IRS
33	Switzerland	0.19	0.33	0.56	IRS	0.19	0.33	0.58	IRS	0.01	0.33	0.03	IRS
34	Nepal	0.44	0.50	0.89	IRS	1.00	1.00	1.00	–	0.03	0.50	0.07	IRS
35	Portugal	0.20	0.30	0.67	IRS	0.16	0.30	0.52	IRS	0.01	0.30	0.02	IRS
36	Ecuador	0.52	0.66	0.79	DRS	0.10	0.46	0.21	IRS	0.05	0.46	0.11	IRS
Mean		0.43	0.53	0.76		0.30	0.52	0.51		0.14	0.53	0.22	
# of efficient DMUs		2	6	10		2	5	2		2	6	2	

*Based on data envelopment analysis efficiency calculated results*.

## References

[1] Newey S Gulland A. What is Coronavirus, How Did It Start and How Big Could It Get? The Telegraph (2020). Available online at: https://www.telegraph.co.uk/news/2020/04/28/what-is-coronavirus-covid-19-virus-pandemic/ (accessed April 28, 2020).

[2] Worldometers. COVID-19 Coronavirus Pandemic. Worldometer (2020). Available onlnie at: https://www.worldometers.info/coronavirus/ (accessed August 08, 2020).

[3] Correia S Luck S Verner E. Pandemics Depress the Economy, Public Health Interventions Do Not: Evidence from the 1918 Flu. Draft Paper. (2020). Available online at: https://ssrn.com/abstract=3561560 (accessed July 01, 2020).

[4] Sarkar K Khajanchi S Nieto JJ. Modeling and forecasting the COVID-19 pandemic in India. Chaos Solit Fractals. (2020) 139:110049. 10.1016/j.chaos.2020.110049 32834603 PMC7321056

[5] Tang B Xia F Bragazzi NL McCarthy Z Wang X He S. Lessons Drawn from China and South Korea for Managing COVID-19 Epidemic: Insights from a Comparative Modelling Study. Bulletin of World Health Organisation (2020). 10.1016/j.isatra.2021.12.004 PMC8713134 35164963

[6] Breitenbach MC Ngobeni V Aye G. Efficiency of healthcare systems in the first wave of COVID-19- a technical efficiency analysis. In: MPRA Paper No. 101440. (2020). Available online at: https://mpra.ub.uni-muenchen.de/101440/ (accessed October 15, 2020).

[7] Khajanchi S Sarkar K. Forecasting the daily and cumulative number of cases for the COVID-19 pandemic in India. Chaos. (2020) 30:071101. 10.1063/5.0016240 32752627 PMC7585452

[8] Samui P Mondal J Khajanchi S. A mathematical model for COVID-19 transmission dynamics with a case study of India. Chaos Solit Fractals. (2020) 140:110173. 10.1016/j.chaos.2020.110173 32834653 PMC7405793

[9] The World Bank. Current Health Expenditure (% of GDP). The World Bank (2020). Available onlnie at: https://data.worldbank.org/indicator/SH.XPD.CHEX.GD.ZS (accessed August 08, 2020).

[10] The World Bank. Nurses and Midwives per 1 000 Population. The World Bank (2020). Available onlnie at: https://data.worldbank.org/indicator/SH.MED.NUMW.P3 (accessed August 08, 2020).

[11] The World Bank. Physicians per 1 000 Population. The World Bank (2020). Available onlnie at: https://data.worldbank.org/indicator/SH.MED.PHYS.ZS (accessed August 08, 2020).

[12] Khajanchi S Sarkar K Mondal J Perc M. Dynamics of the COVID-19 Pandemic in India. arXiv. (2020) arxiv:2005.06286. 10.21203/rs.3.rs-27112/v1

[13] Ngobeni V Breitenbach MC Aye G. Technical efficiency of provincial public healthcare in South Africa. Cost Effect Resour Allocat. (2020) 18:3. 10.1186/s12962-020-0199-y 32002018 PMC6986147

[14] Campanella P Azzolini E Izzi A Pelone F De Meo C La Milia CD. Hospital efficiency: how to spend less maintaining quality. Ann Ist Super Sanita. (2017) 53:46–53. 10.4415/ANN_17_01_10 28361805

[15] Alhassan RK Nketiah-Amponsah E Akazili J Spieker N Arhinful DK De Wit TFR. Efficiency of private and public primary health facilities accredited by the national health insurance authority in Ghana. Cost Effect Resour Allocat. (2015) 13:23. 10.1186/s12962-015-0050-z 26709349 PMC4691298

[16] Jarjue G Nor NM Ghani JA Jalil SH. Technical efficiency of secondary health care service delivery in the Gambia. Int J Econ Manag. (2015) 9:25–43.

[17] Chowdhury H Zelenyuk V Wodchis W Laporte A. Efficiency and Technological Change in Health Care Services in Ontario (No. WP082010). School of Economics, University of Queensland (2010). Available onlnie at: https://economics.uq.edu.au/files/5235/WP082010.pdf (accessed January 01, 2019).

[18] Gannon B. Testing for variation in technical efficiency of hospitals in Ireland. Econ Soc Rev. (2005) 36:273–94.

[19] Marschall P Flessa S. Assessing the efficiency of rural health centres in Burkina Faso: an application of data envelopment analysis. J Public Health. (2009) 17:87. 10.1007/s10389-008-0225-6

[20] Akazili J Adjuik M Jehu-Appiah C Zere E. Using data envelopment analysis to measure the extent of technical efficiency of public health centres in Ghana. BMC Int Health Hum Rights. (2008) 8:11. 10.1186/1472-698X-8-11 19021906 PMC2605432

[21] Masiye F. Investigating health system performance: an application of data envelopment analysis to Zambian Hospitals. BMC Health Serv Res. (2007) 7:58. 10.1186/1472-6963-7-58 17459153 PMC1878476

[22] Zere E Mbeeli T Shangula K Mandlhate C Mutirua K Tjivambi B. Technical efficiency of district hospitals: evidence from Namibia using data envelopment analysis. Cost Effect Resour Allocat. (2006) 4:1. 10.1186/1478-7547-4-5 16566818 PMC1524815

[23] Kirigia JM Sambo LG Scheel H. Technical efficiency of public clinics in Kwazulu-Natal Province of South Africa. East Afr Med J. (2001) 78:1–14. 10.4314/eamj.v78i3.9070 12002061

[24] Kirigia JM Lambo E Sambo LG. Are public hospitals in KwaZulu-Natal Province of South Africa technically efficient? Afr J Health Sci. (2000) 7:25. 17650022

[25] Liu JS Lu LYY Lu WM Lin BJY. A survey of DEA applications. OMEGA. (2013) 41:893–902. 10.1016/j.omega.2012.11.004

[26] Bhat VN. Institutional arrangements and efficiency of healthcare delivery systems. Eur J Health Econ. (2005) 50:215–22. 10.1007/s10198-005-0294-1 15864675

[27] Lo Storto C Goncharuk AG. Efficiency vs. effectiveness: a benchmarking study on european healthcare systems. Econ Sociol. (2017) 10:102–15. 10.14254/2071-789X.2017/10-3/8

[28] Afonso A St Aubyn M. Relative efficiency of health provision: A DEA approach with non-discretionary inputs. In: ISEG-UTL Economics Working Paper (2006).

[29] De Cos PH Moral-Benito E. Determinants of health-system efficiency: evidence from OECD countries. Int J Health Care Finance Econ. (2014) 14:69–93. 10.1007/s10754-013-9140-7 24398651

[30] Hadad S Hadad Y Simon-Tuval T. Determinants of healthcare system's efficiency in OECD countries. Eur J Health Econ. (2013) 14:253–65. 10.1007/s10198-011-0366-3 22146798

[31] Kim Y Kang M. The Measurement of Health Care System Efficiency: Cross-country Comparison by Geographical Region. (2014). Available online at: http://s-space.snu.ac.kr/bitstream/10371/91911/1/02_Younhee_Kim.pdf (accessed January 01, 2019).

[32] González E Cárcaba A Ventura J. Value efficiency analysis of health systems: does public financing play a role? J Public Health. (2010) 18:337–50. 10.1007/s10389-009-0311-4

[33] Santos SP Amado CAE Santos MF. Assessing the efficiency of mother-to-child HIV prevention in low- and middle-income countries using data envelopment analysis. Health Care Manag Sci. (2012) 15:206–22. 10.1007/s10729-012-9196-9 22354634

[34] Shirouyehzad H Jouzdani J Khodadadi-Karimvand M. Fight against COVID-19: a global efficiency evaluation based on contagion control and medical treatment. J Appl Res Indust Eng. (2020) 7:109–20. 10.22105/JARIE.2020.225087.1146

[35] Gavurova B Kocisova K Belas L Krajcik V. Relative efficiency of government expenditure on secondary education. J Int Stud. (2017) 10:329–43. 10.14254/2071-8330.2017/10-2/23

[36] Cooper WW Seiford LM Tone K. Data Envelopment Analysis: A Comprehensive Text with Models, Applications, References and DEA-Solver Software. New York, NY: Springer (2007).

[37] McWilliams A Siegel D Van Fleet DD. Scholarly journals as producers of knowledge: theory and empirical evidence based on data envelopment analysis. Org Res Methods. (2005) 8:185–201. 10.1177/1094428105275377

[38] Aristovnik A. The Impact of ICT on Educational Performance and its Efficiency in Selected EU and OECD Countries: A Non-Parametric Analysis. Elsevier (2012).

[39] Martić M Novaković M Baggia A. Data envelopment analysis-basic models and their utilization. Organizacija. (2009) 42:37–43. 10.2478/v10051-009-0001-6

[40] You S Yan H. A new approach in modeling undesirable output in DEA model. J Operat Res Soc.(2011) 62:2146–56. 10.1057/jors.2011.1

[41] Taylor B Harris G. Relative efficiency among south african universities: a data envelopment analysis. Higher Educ. (2004) 47:73–89. 10.1023/B:HIGH.0000009805.98400.4d

[42] Joumard I André C Nicq C Chatal O. Health Status Determinants: Lifestyle, Environment, Health Care Resources and Efficiency. Elsevier (2008).

[43] Nakashima K Nose T Kuriyama S. A New approach to environmental performance evaluation. Int J Prod Res. (2006) 44:4137–43. 10.1080/00207540600863522

[44] Hua Z Bian Y. DEA with undesirable factors. In: Zhu J, Cook WD, editors. Modelling Data Irregularities and Structural Complexities in Data Envelopment Analysis. Boston, MA: Springer Science (2007). p. 103–21.

[45] Lu WM Lo SF. A benchmark-learning roadmap for regional sustainable development in China. J Operat Res Soc. (2007) 58:841–9. 10.1057/palgrave.jors.2602229

[46] Lu WM Lo SF. A closer look at the economic environmental disparities for regional development in China. Eur J Operat Res. (2007) 183:882–94. 10.1016/j.ejor.2006.10.027

[47] Dyckhoff H Allen K. Measuring ecological efficiency with data envelopment analysis (DEA). Eur J Operat Res. (2001) 132:312–25. 10.1016/S0377-2217(00)00154-5

[48] Golany B Roll Y. An application procedure for DEA. Omega. (1989) 17:237–50. 10.1016/0305-0483(89)90029-7

[49] Seiford LM Zhu J. Modelling undesirable factors in efficiency evaluation. Eur J Operat Res. (2002) 142:16–20. 10.1016/S0377-2217(01)00293-4

[50] Färe R Grosskopf S Lovell CAK Pasurka C. Multilateral productivity comparisons when some outputs are undesirable: a nonparametric approach. Rev Econ Stat. (1989) 71:90–8. 10.2307/1928055

[51] Zhou P Ang BW Poh KL. A mathematical programming approach to constructing composite indicators. Ecol Econ. (2007) 62:291–7. 10.1016/j.ecolecon.2006.12.020

[52] Yang C Hsiao C Yu M. Technical efficiency and impact of environmental regulations in farrow-to-finish swine production in Taiwan. Agric Econ. (2008) 39:51–61. 10.1111/j.1574-0862.2008.00314.x

[53] Stewart H Proctor K Siddique H. Johnson: Many More People Will Lose Loved Ones to Coronavirus. (2020). Available online at: https://www.theguardian.com/world/2020/mar/12/uk-moves-to-delay-phase-ofcoronavirus-~plan (accessed June 11, 2020).

[54] Watts J. Delay is Deadly: What Covid-19 Tell Us About Tackling the Climate Crisis. (2020). Available online at: Governments-Coronavirus. https://www.theguardian.com/commentisfree/2020/mar/24/covid-19-climatecrisis- (accessed June 11, 2020).

